# Brassinosteroids in Cucurbits: Modulators of Plant Growth Architecture and Stress Response

**DOI:** 10.3390/ijms26157234

**Published:** 2025-07-26

**Authors:** Renata Słomnicka, Magdalena Cieplak, Ana Montserrat Martín-Hernández, Grzegorz Bartoszewski

**Affiliations:** 1 Department of Plant Genetics, Breeding and Biotechnology, Institute of Biology, Warsaw University of Life Sciences, Nowoursynowska 159 Street, 02-776 Warsaw, Poland; renata_slomnicka@sggw.edu.pl (R.S.); magdalena_cieplak@sggw.edu.pl (M.C.); 2 Centre for Research in Agricultural Genomics (CRAG) CSIC-IRTA-UAB-UB, C/Vall Moronta, Edifici CRAG, Bellaterra (Cerdanyola del Vallés), 08193 Barcelona, Spain; montse.martin@irta.cat; 3 Institut de Recerca i Tecnologia Agroalimentàries (IRTA), Edifici CRAG, Campus UAB, Bellaterra, Cerdanyola del Vallès, 08193 Barcelona, Spain

**Keywords:** crop improvement, crop productivity, dwarfism, plant development, plant hormones, precision breeding

## Abstract

Brassinosteroids (BRs) are steroid hormones that are essential for plant growth, development, and environmental adaptation. They control the division, elongation, and differentiation of various cell types throughout the entire plant life cycle, affecting growth and the stress response. Therefore, fine-tuning of BR biosynthesis and modulation of signaling pathways offer possibilities for developing cultivars characterized by adjusted plant architecture or improved stress tolerance to benefit crop production. Additionally, precise BR treatments can be employed to increase the productivity of crop plants. This review aims to provide a comprehensive summary of the genetic basis of traits related to BR metabolism and signaling in cucurbits, the second largest vegetable family, which contributes significantly to global vegetable production and nutritional security. We summarize the current knowledge concerning BR biosynthesis mutants, the role of BRs in stress mitigation, and the potential of the exogenous application of BRs to alleviate stress during cucurbit production. We also discuss how genes related to BR metabolism can be used to develop gene editing strategies to advance precision breeding in cucurbits.

## 1. Introduction

The Cucurbitaceae family contains nearly 1000 species in more than 100 genera of plants growing in tropical and subtropical climate zones [1]. After a complex domestication history, which started 11,000 years ago in the New World and Asia and later in Africa, eight cucurbit species are now cultivated worldwide and are of economic importance. The major cultivated cucurbits include cucumbers (*Cucumis sativus* L.), watermelons (*Citrullus lanatus* (Thunb.) Matsum. & Nakai), melons (*Cucumis melo* L.), pumpkins and squashes (*Cucurbita pepo* L. *Cucurbita moschata* Duchesne, *Cucurbita maxima* Duchesne), bitter gourds (*Momordica charantia* L.), and bottle gourds (*Lagenaria siceraria* (Molina) Standl.). There is also a group of approximately 20 cucurbit species of local commercial importance cultivated in their native regions [2]. The global production of cucurbits in 2023 was estimated to be approximately 250 million tons, and the cultivation area was 8 million hectares [3]. Cucurbits play an important role in the human diet by providing nutrients such as vitamins and minerals. Depending on the specific crop, the fruits of cucurbits can be eaten fresh or processed. Cucurbit seeds are also consumed. In addition to being consumed by humans, fruits are used as components of animal fodder or ornamentals. Some cucurbit fruits, such as bitter gourd, have medicinal properties [4]. In addition, the fruits or seeds of particular species have potential applications in the cosmetic industry [5].

In plants, one of the most impactful domestication traits across crops has been altered plant architecture. Domestication syndrome is often associated with a change toward more compact growth while maintaining a satisfactory yield [2]. Plant architecture refers to the morphological characteristics and spatial distribution of the organization of the part of a plant that is above ground. It is an important agronomic trait in plant breeding that affects crop growth, yield, and stress resilience [6,7]. Currently, one of the major challenges in plant breeding is developing cultivars with improved plant architecture to secure crop yields under different growth conditions. The altered compactness of plants affects the distribution of solar radiation in the population, carbon assimilation, and dry matter accumulation, which can ultimately result in increased yields per cultivation area. It also directly determines the workload in crop management and harvesting [8,9,10]. Dwarf or semi-dwarf cultivars are also suitable for intercropping and, in turn, improve the index of multiple cropping, and increase yields per unit area [11]. Owing to their impact on crop management, dwarfism or semi-dwarfism is an important agronomic trait in cucurbit breeding. To date, the potential of this type of plant architecture seems underexplored in these crops. Thus, there is an effort to identify and characterize genes that control plant growth architecture. Over 30 genes have been identified as shaping plant architecture in cucurbits [12]. Some of these genes have been fine-mapped and characterized, and some of them are involved in the metabolism of brassinosteroids (BRs).

This review aimed to (A) summarize the current progress in elucidating the genetic basis of dwarfism in cucurbits related to genes involved in BR metabolism, (B) highlight the role of BRs in the abiotic and biotic stress responses of cucurbits, and (C) explore the possibility of applying exogenous BRs to improve cucurbit production. The potential of gene editing as a tool for precision breeding to develop BR-based strategies to increase cucurbit productivity was also considered.

## 2. Chemical Structure and Occurrence of BRs

Brassinosteroids are an important class of steroidal plant hormones. They are present at extremely low concentrations in the cells of all plant tissues at every stage of development. Endogenous BRs have been detected in stems, roots, leaves, anthers, pollen, flowers, and seeds [13,14]. The richest sources of BRs are pollen grains and immature seeds, while their lower concentrations are detected in shoots and leaves. The concentration of BRs in young tissues is generally greater than that in mature tissues [15]. The first brassinosteroid discovered in the pollen of rapeseed (*Brassica napus* L.) was brassinolide (BL) [16]. To date, more than 69 BRs have been identified in 64 plant species, constituting 53 angiosperms [14,17]. However, only 52 BRs have been characterized as having biological activities in plants. The most common BRs are BL, castasterone (CS), 6-deoxocastasterone (6-deoxoCS), 28-norcastasterone (28-norCS), teasterone (TE), and typhasterol (TY) [15].

**Figure 1:**
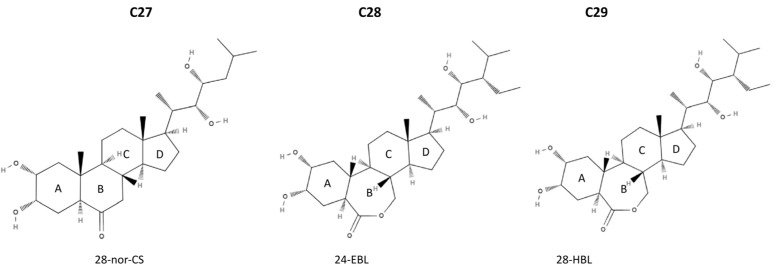
Chemical structures representing major types of brassinosteroids (BRs): C27: 28-norcastasterone (28-nor-CS), C28: 24-epibrassinolide (24-EBL), and C29: 28-homobrassinolide (28-HBL) with marked A, B, C, and D rings. Structures were adapted from PubChem (accessed on 15 July 2025; CIDs: 13982110, 4430550, and 11038340, respectively).

BRs are divided into C27, C28, and C29 types on the basis of the total number of carbon atoms and differ in the presence or absence of alkyl groups (Figure 1). The basic carbon skeletons are 5α-cholestane for C27 BRs, 5α-ergostane for C28 BRs, and 5α-stigmastane for C29 BRs. C27-BRs do not carry an alkyl group. C28-BRs include 24-methylene, 24S-methyl, and 24R-methyl BRs. C29-BRs contain 24-ethylidene, 24-ethyl, and 24-methylene-25-methyl BRs [13]. C27-BRs have lower biological activity and occurrence. For this reason, minimal attention has been given to their physiological value in plants [18]. Due to their stronger biological activity and wider distribution in the plant kingdom, CS and BL are the most important bioactive BRs in plants, classified as C28-BRs, and have been extensively studied for their biosynthesis by means of feeding experiments as well as the molecular genetics of BR-deficient mutants [18,19]. The C29-BR biosynthesis pathway is likely linked to the C-28-BR biosynthesis pathway to maintain biologically active BRs in plants [20]. All natural BRs share a 5α-cholestane skeleton, and their structural differences arise from the type and orientation of the oxygen-containing functional groups on rings A and B (Figure 1) [21,22,23,24]. In addition, BRs conjugated with sugars or fatty acids have been identified in plants [13]. The structural diversity of BRs may influence their properties and complicate their purification, identification, and quantification [25,26].

## 3. BR Biosynthesis and Signaling

To date, numerous studies have investigated the metabolism of BRs. The first report of the biosynthesis of BL and its analogs was published by Wada and Marumo in 1981 [27]. The biosynthesis of BRs is highly complex. The triterpene squalene is a precursor for sterol biosynthesis, where it is cyclized to cycloartenol through a series of reactions involving sterol delta-7 C5 desaturase (DWF7), sterol delta-7 reductase (DWF5), and sterol C24 reductase (DWF1). The final steps of the sterol biosynthetic pathway involve the isomerization and reduction of 24-methylene cholesterol (24-MC) to campesterol (CR) [28]. The primary biosynthesis of BRs begins from CR and proceeds through parallel and highly interconnected pathways. CR is first converted into campestanol (CN) via the late C22 oxidation pathway. CN is converted to CS via the early or late C6 oxidation pathway, which is collectively called the CN-dependent pathway [21]. In the alternative CN-independent pathway, CR is converted into 6-deoxoCS via the early C22 oxidation pathway, either directly or through C23 hydroxylation. The path then continues through C6 oxidation steps to synthesize CS [14,29]. CS is subsequently converted to BL via Baeyer–Villiger oxidation in *Arabidopsis* and tomato but not in rice [30,31,32].

**Figure 2:**
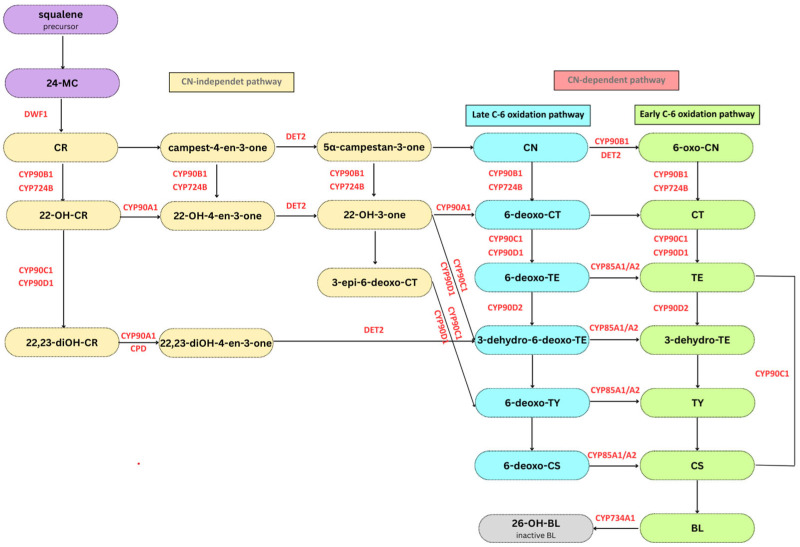
Schematic representation of the brassinosteroid (BR) biosynthesis pathway (modified from [19,34,35]). BR biosynthesis begins with squalene (violet background), and many BR intermediates are subsequently synthesized (black letters) to produce biologically active BRs. BR biosynthesis is catalyzed by numerous enzymes (red letters). The process consists of two pathways: the CN-independent pathway (yellow background) and the CN-dependent pathway, which includes early (green background) and late C6-oxidation (blue background). The CYP734A1 inactivates BL into 26-OH-BL (gray background) via C26 hydroxylation to maintain optimal hormone levels.

Numerous enzymes participate in BR biosynthesis, including the steroid-5-alpha reductase DEETIOLATED 2 (DET2, DWF6), C22 hydroxylases DWARF4 (CYP90B1, DWF4) and DWARF11 (CYP724B1, DWF11), C23 hydroxylases ROTUNDIFOLIA 3 (CYP90C1, ROT3) and CYP90Ds, C3 oxidase CONSTITUTIVE PHOTOMORPHOGENIC DWARF (CYP90A1, CPD), C26 hydroxylase PHYB ACTIVATION TAGGED SUPPRESSOR 1 (CYP734A1, BAS1), BRASSINOSTEROID-6-OXIDASE 1 (CYP85A1, BR6OX1), and BRASSINOSTEROID-6-OXIDASE 2 (CYP85A2, BR6OX2) (Figure 2) [14,29,33,34,35]. Loss-of-function mutations of the genes encoding these enzymes lead to similar plant phenotypes in different species: dwarfism, dark green leaves, shorter roots, delayed flowering, reduced biomass, and seed yield [34]. Modulating the activity and level of key enzymes in the BR biosynthetic pathway is a way to alter BR levels. Additionally, the exogenous application of BL led to the downregulation of BR biosynthetic genes. BR biosynthesis is subject to feedback inhibition by its end products, CS and BL. Conversely, inhibitors of BR biosynthesis upregulate these genes, indicating that feedback transcriptional regulation occurs at multiple points along the pathway [36,37].

**Figure 3:**
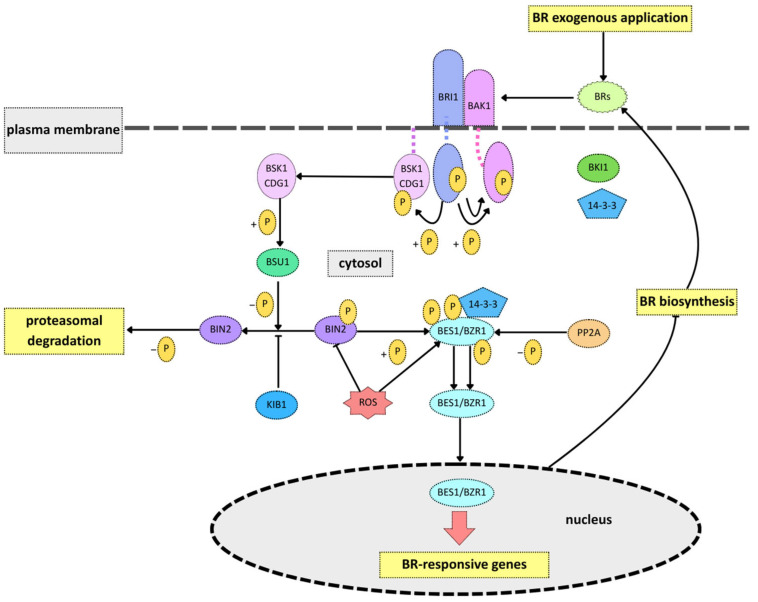
A general scheme of the brassinosteroid (BR) signaling pathway from recognition to the transcriptional activation of BR-responsive genes in the nucleus (based on [35,40,46]). BRs are recognized by a receptor complex containing BRI1 and BAK1. The binding of BR receptor kinases triggers the dephosphorylation and accumulation of the nuclear proteins BZR1/BES1, which is presumably achieved by inhibiting the negative regulator BIN2. In the absence of BR, the BIN2 kinase phosphorylates and targets BZR1/BES1 for degradation via the ubiquitin-dependent proteasome pathway.

The signal transduction pathway of BRs has been intensively studied and is recognized as a complex regulatory network (Figure 3). The BR signaling cascade can be categorized into three significant steps: (1) recognition of BRs and early activation of the receptor kinase BRASSINOSTEROID INSENSITIVE 1 (BRI1) and its coreceptor BRI1-ASSOCIATED KINASE 1 (BAK1); (2) inactivation of negative regulators such as BRASSINOSTEROID INSENSITIVE 2 (BIN2) and associated phosphatases and kinases; and (3) regulation of transcription factors, including BRI1-EMS-SUPPRESSOR 1 (BES1) and BRASSINAZOLE-RESISTANT 1 (BZR1) [28]. Upon BR binding, BRI1 and BAK1 are activated, triggering a phosphorylation cascade that includes BRI1 KINASE INHIBITOR 1 (BKI1), a negative regulator of BR signaling [38,39]. Phosphorylation leads to the dissociation of BKI1 from the plasma membrane and its subsequent interaction with 14-3-3 proteins, which play a role in the cytoplasmic retention of BES1 and BZR1 [40]. Simultaneously, activated BRI1 phosphorylates BR-SIGNALING KINASE 1 (BSK1) and CONSTITUTIVE DIFFERENTIAL GROWTH 1 (CDG1), which together activate BRI1 SUPPRESSOR 1 (BSU1), a phosphatase that dephosphorylates and inactivates BIN2, a GSK3-like kinase and a key repressor of BR signaling [41,42,43]. The inactivation of BIN2 is further regulated by KINK SUPPRESSED IN BZR1-1D (KIB1), which prevents its interaction with BZR1/BES1 and promotes its ubiquitination and degradation. Once BIN2 is inactivated, the key transcription factors BZR1 and BES1 are rapidly dephosphorylated by PROTEIN PHOSPHATASE 2A (PP2A) and dissociate from the 14-3-3 proteins. This enables their translocation into the nucleus, where they regulate the expression of BR-responsive genes [44]. The regulation of transcription factors, including BES1 and BZR1, inhibits BR biosynthesis. In contrast, in the absence of BRs, BKI1 binds to the intracellular domain of BRI1, blocking its interaction with BAK1 and thereby inhibiting downstream signaling [38]. Under these conditions, BIN2 remains active and phosphorylates BZR1 and BES1. The phosphorylated forms of these transcription factors have reduced DNA-binding affinity and limited nuclear accumulation. Additionally, their association with 14-3-3 proteins results in their retention in the cytoplasm, preventing their function as transcriptional regulators [45].

## 4. Functions of BRs in Plants

**Figure 4:**
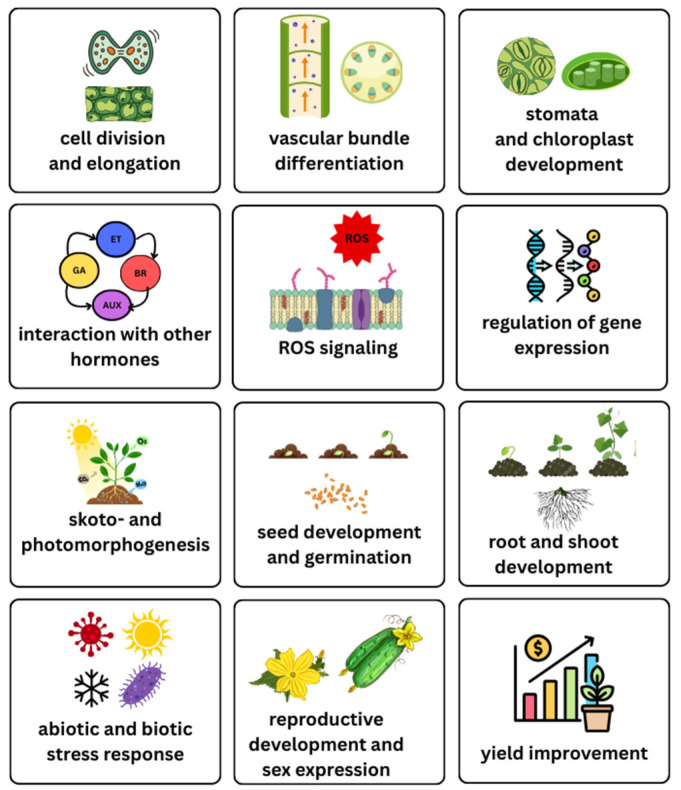
Roles of brassinosteroids (BRs) in crop plants.

BRs play crucial roles in the growth and development of plants (Figure 4) [40,47]. They take part in the regulation of diverse developmental and physiological processes, including cell division, stem cell maintenance, vascular tissue development, the elongation of different cell types, and floral transition [47,48,49]. They can also regulate hypocotyl elongation, root and shoot growth, stomatal patterning, tracheary element differentiation, xylem formation and differentiation, photo- and skotomorphogenesis, and plant senescence [46]. For example, BRs promote stem cell daughter division in the primary root meristem, as revealed by comparing the small meristem of BR-deficient mutants with that of the wild type. In contrast, high levels of BRs increase meristem differentiation in roots and may participate in optimal cell cycle control [50]. Another study suggested that BRs partially control three separate functions: cell division, the rate of cell elongation, and the termination of cell elongation [51]. In *Arabidopsis*, BIN2 has been shown to interact with tubulin and, through its effect on microtubules, directly regulates pavement cell development and, consequently, organ elongation [52]. On the other hand, the inactivation of BIN2 causes the activation of the MAPK pathway, resulting in reduced stomatal development [53].

Kelly-Bellow et al. [54] revealed that DWF4 is essential for maintaining the turgor balance between cell layers and maintaining normal growth. In turn, the differentiation of cambium cells into xylem vessels is dependent on GSK3-like kinases [55]. Interestingly, loss-of-function mutants of BZR1 or BES1 present reduced xylem differentiation [56]. BIN2-BES1 was also found to regulate cambial activity and promote phloem differentiation [57,58].

BRs are involved in plant reproduction, including pollen development, pollen tube growth, and seed germination [46]. They also play a vital role in regulating male and female fertility [59,60]. Mutants in genes associated with BR signaling exhibit male sterility due to a defective or omitted layer of tapetal cells in their anthers, suggesting that members of the BES1/BZR1 family are essential for tapetum development [61]. In maize, the *nana plant 1* (*na1*) mutant, which carries a loss-of-function mutation in a DET2 ortholog, contains feminized male flowers with a tassel-seed phenotype [59]. BR-promoted pollen and seed development in rice are achieved by stimulating the expression of CARBON STARVED ANTHER (CSA), which triggers the expression of sugar partitioning and metabolic genes through OsBZR1 [62]. In BR-deficient mutants, stigma elongation is retarded, the anther fails to release pollen grains, and the microspores are vacuolated and degenerated [63].

BRs are also involved in the regulation of plant architecture, tiller number, leaf angle, and leaf size [46]. For example, BR-deficient and BR-insensitive mutants present dwarf phenotypes with compact and wrinkled leaves [64]. In contrast, BR gain-of-function mutants present elongated organs [65]. Some BR signaling gain-of-function mutants, such as *bzr1-1D*, also display organ-fusion phenotypes [66]. In *Arabidopsis*, auxin response factors (ARFs) regulate DWF4 expression and control leaf shape by promoting homogalacturonan (HG) dimethyl-esterification [67]. In turn, tomato SlBZR1 binds to the promoter of BRANCHED1 (BRC1), a TCP family transcription factor; negatively regulates the outgrowth of buds; and suppresses their expression, promoting shoot branching [68].

Recent studies have provided strong evidence that BRs play a role in coordinating growth and defense in many plant species by increasing plant tolerance/resistance to a wide range of stresses caused by drought, extreme temperatures, salinity, flooding, heavy metals, oxidative stress, and pathogens [22,35,46,69]. In particular, the regulation of BES and BZR expression and their interaction with stress-responsive genes can promote stress tolerance in plants [70]. For example, the accumulation of BZR1 and BES1 promotes the expression of C-REPEAT/DEHYDRATION-RESPONSIVE ELEMENT BINDING FACTOR1 (CBF1) and CBF2, which positively regulate cold stress responses in *Arabidopsis* [71]. In contrast, *AtBES1* also enhances heat stress tolerance by interacting with the heat shock transcription factor HSFA1 [72]. The interaction of *AtBES1* with drought-responsive TFs, including RD26 and WRKY54, results in the negative regulation of drought tolerance [73,74]. In turn, *TaBZR2* in wheat and *ZmBES1/BZR1-5* in maize positively regulate drought stress tolerance [75,76]. Many studies have revealed that the application of exogenous BRs also plays a role in the development of tolerance to biotic and abiotic stresses. This role includes increased photosynthesis and biomass, increased antioxidant enzymes and detoxification capacity, and increased expression of related genes [46]. These findings underscore the central role of BRs as master regulators of plant growth, reproduction, and stress adaptation. The integration of BRs with other hormonal and environmental signals positions BRs as promising targets in crop improvement strategies.

## 5. Crosstalk Between BRs and Other Phytohormones

BRs interact synergistically and antagonistically with other phytohormones, including ethylene (ET), gibberellins (GAs), and auxins (AUXs), to coordinate various developmental and stress-response processes. These interactions are particularly relevant in cucurbits, where ET and GAs play important roles in flower differentiation and sex development; however, they are still not sufficiently understood.

### 5.1. Interaction Between BRs and Ethylene

**Figure 5:**
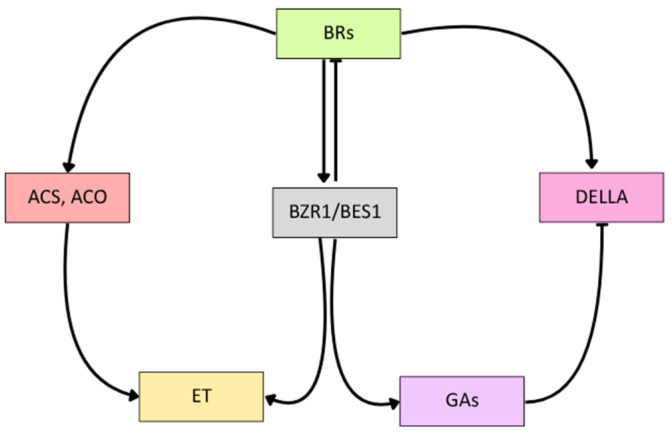
Scheme of the crosstalk between brassinosteroids (BRs) and other phytohormones, ethylene (ET) and gibberellins (GAs).

BRs interact with ET to regulate the gravitropic response of the shoot and are involved in ET-controlled processes in the hypocotyls of light- and dark-grown *Arabidopsis* seedlings [77]. The interaction between BRs and ET occurs at two levels, in which BRs regulate ET production at the transcriptional and posttranscriptional levels (Figure 5). BRs affect ET biosynthesis mostly by regulating the activities of 1-aminocyclopropane-1-carboxylic acid (ACC) synthase (ACS) and ACC oxidase (ACO), as shown in *Arabidopsis* [78]. In cucumber, the interaction between BRs and ET has been studied in the context of sex expression and the abiotic stress response [79,80,81]. The application of 24-epibrassinolide (EBL) caused a significant decrease in the time of appearance of the first female flower in a monoecious cucumber. Additionally, it increased the number of female flowers on the main stem. EBL application also increased ET production in cucumber seedlings, suggesting that the effect of BRs may be mediated by ET [79]. In cucumber, the alleviative effects of EBL and ACC on seed germination in the presence of NaCl were observed. EBL attenuated the reduction in ET production from imbibed seeds caused by salt stress. Moreover, the addition of EBL reversed the decrease in ACO activity triggered by salt stress. On the other hand, the alleviatory effect of EBL on seed germination in the presence of NaCl was attenuated by the antagonist of ET synthesis, aminoethoxyvinylglycine (AVG). Together, these findings suggest that BRs mitigate the effects of salt stress on seed germination through ET-mediated pathways [80].

BL alleviated stress-induced oxidative damage in cucumber seedlings and significantly increased ET biosynthesis and mitochondrial alternative oxidase (AOX) activity. The transcript levels of ET biosynthesis genes (*CsACS1*, *CsACS2*, *CsACS3*, *CsACO1*, and *CsACO2*) and *CsAOX* increased after BL treatment. The application of an ET biosynthesis inhibitor (aminooxyacetic acid, AOA) and an AOX inhibitor (salicylhydroxamic acid, SHAM) suppressed BR-induced alternative respiration and reduced stress tolerance. A hypothetical model describing the crosstalk between BL, ET, and reactive oxygen species (ROS) in BL-induced alternative respiration was proposed: BRs induce ET and ROS production and subsequently increase AOX activity; next, AOX eliminates excess ROS, reduces oxidative damage, and improves stress tolerance [81].

### 5.2. Interaction Between BRs and Gibberellins

Studies from independent research groups revealed evidence of a cooperative and interdependent relationship between BRs and GAs with multiple layers of this interaction (Figure 5). The interaction of the BZR1 transcription factor and DELLA proteins mediates direct crosstalk between BRs and GAs in regulating cell elongation in *Arabidopsis* [82,83,84]. Both GAs and BRs affect the expression of *BZR1* [85,86]. Kang et al. [87] revealed a multifaceted regulatory mechanism of BR and GA cooperation in response to pathogen infection in cucumbers. The knockout and overexpression of the *CsCYP85A1* gene, which is involved in BR biosynthesis, reduced the endogenous GA level during *Phytophthora melonis* infection. The silencing of genes encoding key enzymes involved in GA biosynthesis, ent-kaurenoic acid oxidase (KAO) and GA20 oxidase 1 (GA20ox1), suppressed BR-induced resistance, indicating that proper GA homeostasis is essential for the BR-mediated immune response. Moreover, BZR6, a key regulator of BR signaling, was found to interact physically with GA20ox1, thereby suppressing its expression. Moreover, the silencing of *BZR6* promoted endogenous GA biosynthesis and compromised GA-mediated cucumber resistance to *P. melonis* [87].

### 5.3. Interaction Between BRs and Auxins

Numerous studies have confirmed the mutual interaction between BRs and AUXs in various plant development processes; however, the interaction between BRs and AUXs in cucurbits has not been extensively studied [40]. The application of 28-homobrassinolide (HBL) and indole-3-acetic acid (IAA) synergistically increased the AUX-induced elongation of cucumber hypocotyls. These observations led to the conclusion that BRs increase AUX activity and possess growth-promoting activity [88]. In other studies, decreased expression of the *CsARF10a* gene was observed in cucumber ovaries after BR treatment, which stimulated the formation of parthenocarpic fruits [89].

Taken together, these findings illustrate that BRs integrate with multiple hormone signaling pathways—including ET, GAs, and AUX—to regulate diverse biological processes, from seed germination to stress tolerance. While emerging studies in cucumber provide valuable insights, further research is needed to dissect the spatiotemporal dynamics of these interactions in other cucurbits.

## 6. Mutants in Cucurbits with Affected BR Biosynthesis

The plant architecture of cucurbits is generally determined by multiple traits, such as determinate and indeterminate growth habits, shoot branching and/or vine length, leaf morphology, and tendrils [12].

In cucumber, several *super compact* (*scp*) or *compact* (*cpa*) mutants have been described; however, only a few have been characterized at the molecular level. In cucumber, the candidate genes of the five mutants (*scp-1*, *scp-2*, *scp-3*, *cpa*, and *cpa-2*) have been demonstrated to be involved in BR biosynthesis (Table 1). The phenotypes of all of these mutants—characterized by shortened internodes and petioles, the absence of lateral branches, and darkened, wrinkled leaves—are controlled by single recessive genes [90,91,92,93,94].

Map-based cloning identified the *CsCYP85A1* gene on chromosome 5 as a candidate for *scp-1*. CsCYP85A1 encodes a BR C6-oxidase in the BR biosynthesis pathway. A G-to-A transition in the second exon resulted in the substitution of a tryptophan (TGG) with a stop codon (TAG) at position 157 of the mutated protein. Three copies of *CsCYP85A* are present in the cucumber genome, but only *CsCYP85A1* is active. *CsCYP85A1* is expressed in all tissue types but is more highly expressed in flowers than in leaves and stems [90]. Other C6-oxidases, encoded by *CYP85A* genes, have been identified in *Arabidopsis* (*AtCYP85A1* and *AtCYP85A2*), tomatoes (*SlCYP85A1* and *SlCYP85A3*), peas (*PsCYP85A1* and *CYP85A6/LKE*), and grapes (*VvCYP85A1*) [34]. Interestingly, all CYP85A1 proteins mediate the conversion of 6-deoxo-CS to CS, whereas CYP85A3 in tomato and CYP85A2 in *Arabidopsis* convert 6-deoxo-CS into BL via CS [30,31]. Additionally, tomato *SlCYP85A2* is expressed only in fruits, which correlates with the presence of BL in tomato fruits. For this reason, the tomato mutant *d^x^* presents a strongly dwarf phenotype without negatively affecting fruit production [31]. In *Arabidopsis*, the *cyp85a1* mutant does not present a mutant phenotype, and the *cyp85a2* mutant has a weak dwarf phenotype due to the functional redundancy of the *AtCYP85A1* and *AtCYP85A2* genes, which are expressed in all tissue types [30]. Only the *cyp85a1cyp85a2* double mutant displays a severe dwarf phenotype in *Arabidopsis* [31].

The cucumber *scp-2* mutant was shown to be associated with the *CsDET2* gene on chromosome 3, encoding the steroid 5-alpha reductase, which is a key early step enzyme in BR biosynthesis. Two transitions and a single nucleotide insertion were found in the coding region of the mutant allele. The insertion resulted in protein truncation and a lack of 29 aa in the C-terminus, affecting the DET2 reductase domain [91]. In species such as *Arabidopsis*, maize, pea, and cotton (except soybean) *DET2* has been reported to be encoded by a single gene [29,34]. All the *det2* mutants in these species exhibit developmental and growth defects including extreme dwarfism, reduced apical dominance, dark green leaves, and reduced fertility, similar to the *scp-2* mutant [34,91]. Recently, the cucumber *scp-3* gene was characterized. BSA-seq combined with map-based cloning allowed the identification of a T-to-A transversion within the second exon of the *CsDWF7* gene, which is located on chromosome 4. This mutation leads to the substitution of leucine (CTT) with histidine (CAT) at position 156 of the protein (L156H). Compared with the wild type, *scp-3* presented reduced *CsDWF7* expression in various tissues [94]. To date, the *DWF7* gene has not been described in any plant species other than *Arabidopsis*. However, on the basis of phylogenetic analysis, Zebosi et al. [29] reported that in *Arabidopsis,* maize, and soybean, *DWF7* consists of duplicate pairs, in contrast to rice and *Brachypodium*, which have only one copy. Both cucumber and *Arabidopsis* mutants of the *DWF7* gene present extremely dwarf phenotypes; however, the fertility of *scp-3* plants is more severely reduced than that of *Arabidopsis* mutants [94]. The skotomorphogenic response of all three mutants, *scp-1*, *scp-2*, and *scp-3*, was affected, and they presented noticeably shorter hypocotyls than the wild-type seedlings. Interestingly, some differences were observed in cotyledon opening in etiolated mutant seedlings only. Moreover, in all three mutants, exogenous application of BL only partially restored the phenotype [90,91,94].

The cucumber dwarf mutants *cpa* and *cpa-2* are impaired in sterol biosynthesis, which is a prerequisite for the biosynthesis of BRs. BSA-seq combined with genetic mapping revealed that the *CsDWF5* gene on chromosome 7 is a candidate for *cpa* and that *CsDWF1*, which is also located on chromosome 7, is a candidate for *cpa-2* [92,93]. *CsDWF5* encodes a 7-dehydrocholesterol reductase and the G-to-A transition in the splicing site, causing a 3 bp insertion (TAG), which was found in the first base of the sixth intron of *CsDWF5*, which further resulted in a frameshift mutation and a premature stop codon. The expression of *CsDWF5* is downregulated in different tissues [92]. The candidate gene for *cpa-2*, the *CsDWF1* gene, encodes a C24 reductase and a G-to-A transition in the second exon, resulting in a glutamic acid (GAA)-to-lysine (AAA) substitution at the 502 position of the protein (E502K). A lower expression of *CsDWF1* in *cpa-2* was observed [93]. Like those of *scp* mutants, both the *cpa* and *cpa-2* phenotypes can be only partially restored by exogenous BR application [92,93]. The *DWF5* gene has not been characterized in most species except *Arabidopsis*, in which it occurs as a single copy [29,95]. In addition, the *Arabidopsis dwf5-1* mutant is the only BR mutant in which no decrease in fertility is observed [95]. The *DWF1* gene is present in a single copy in *Arabidopsis* and rice but in two copies in corn, soybean, and *Brachypodium* [29]. Similar to cucumber *cpa-2*, mutants of the *DWF1* gene display dwarfism or semi-dwarfism with reduced fertility in *Arabidopsis*, maize, and rice [29]. In soybean, the *Gmdwf1a* and *Gmdwf1b* mutants present a more severe dwarf phenotype in the double mutant; however, the single *Gmdwf1a* mutant produces significantly more pods and seeds than wild-type plants [96].

The first BR-deficient mutant of *C. pepo*, *dwfcp*, was recently characterized in zucchini [97]. A G-to-A transition in the *Cp4.1LG17g04540* gene, the zucchini ortholog of *DWF5*, leads to a premature stop codon and the truncation of the 7-dehydrocholesterol reductase CpDWF5, a key enzyme in BR biosynthesis. The *dwfcp* mutant presented reduced expression of *CpDWF5* and decreased BL content in most of the investigated organs. Partial rescue of the phenotype was achieved through exogenous BL application. During germination and the early stages of seedling development, the *dwfcp* mutant was less affected by salt stress, and simultaneously, increased expression of genes associated with salt tolerance was observed. The results revealed that in zucchini, *CpDWF5* is a positive regulator of plant growth and a negative regulator of salt tolerance [97].

Another mutant of zucchini *tin4* was reported by Asensio et al. [98] with the candidate gene *CpTINY4*, an ortholog of *SOMATIC EMBRYOGENESIS RECEPTOR-LIKE KINASE* (*SERK*), which encodes a protein kinase with leucine-rich repeats (LRR-RLK) located in the plasma membrane. SERK and two other LRR-RLK proteins, BRI1 and BAK1, form a complex for BR perception. The *tin4* mutant results in severe compaction of vegetative organs caused by the reduced petiole size of leaves and stems, which is akin to the phenotype of the *Arabidopsis bri1*/*serk* double mutant. New loss-of-function alleles of this gene are being generated via gene editing [98].

In watermelon, a dwarf mutant resulting from the insertion of transferred DNA (T-DNA), which displays growth retardation throughout development with shorter internodes and smaller leaves, has been reported [99]. This comprehensive analysis allowed the identification of the *ClDUF21* gene associated with BR biosynthesis. CRISPR/Cas9-mediated knockout of *ClDUF21* resulted in a pronounced dwarf phenotype. Subsequently, CRISPR/Cas9 was used to knock out the homolog of *CsDUF21* in cucumber, and the protein interaction between ClDUF21 and ClDWF1 was confirmed. In the watermelon *Clduf21* loss-of-function mutant, stem insensitivity to exogenous BL was observed. Additionally, the exogenous application of brassinazole (BRZ), an inhibitor of BR biosynthesis, significantly reduced the internode length, delayed growth, and elevated the chlorophyll content in the leaves of the wild-type plants, similar to what was observed in the *Clduf21* mutant. These findings suggest a possible interaction between ClDUF21 and ClDWF1, highlighting the active role of ClDUF21 in BR biosynthesis and its effect on plant height [99].

**Table 1:** Cucurbit mutants related to altered BR biosynthesis.

Species	Mutant	Phenotype	Candidate Gene	Gene ID *	Gene Annotation	Reference
*Cucumis sativus*	*cpa* compact plant architecture	extreme dwarf phenotype shortened internodes and petioles dark green and wrinkled leaves	*CsDWF5*	CsaV3_7G033720	7–dehydrocholesterol reductase	[92]
	*cpa–2* compact plant architecture 2	compact phenotype short stem with few branches shortened internodes and petioles dark green and wrinkled leaves short hypocotyl abnormal stigma and ovary	*CsDWF1*	CsaV3_7G030530	sterol-C24-reductase	[93]
	*scp–1* super compact–1	extremely reduced internodes and mature vine length dark green and wrinkled leaves with a rounder shape no tendrils smaller root length and volume abnormal stigma and ovary de-etiolation in the dark	*CsCYP85A1*	CsaV3_5G038650	BR-6-oxidase	[90]
	*scp–2* super compact–2	extreme dwarf phenotype shortened petioles dark green and wrinkled leaves dark green cotyledons short and inflated hypocotyl de-etiolation in the dark defects in cell elongation and vascular development partially female sterile	*CsDET2*	CsaV3_3G034190	steroid-5-alpha reductase	[91]
	*scp–3* super compact–3	shortened internodes dark green and wrinkled leaves	*CsDWF7*	CsaV3_4G028790	delta7-sterol C5-reductase	[94]
*Cucurbita pepo*	*dwfcp*	shortened internodes dark green and wrinkled leaves shorter and thicker roots greater root biomass reduced fertility	*CpDWF5*	Cp4.1LG17	7–dehydrocholesterol-reductase	[97]
*Citrullus lanatus*	*dwarf*	dwarf phenotype shortened internodes smaller leaf area lacking clear lobulations	*ClDUF21* *ClDWF1*	Cla97C06G115300 Cla97C09G166970	DUF21 domain protein sterol-C24-reductase	[99]

* Gene ID and genome locations refer to the following reference genomes: cucumber to 9930 v3, *C. pepo* MU-CU-16 v4.1 and watermelon 97103 v2.5.

## 7. Role of BRs in the Development and Growth of Cucurbits

BRs take part in the regulation of the growth and development of plants, maintaining BR homeostasis, and allowing plants to adapt to environmental conditions. In many species, the application of exogenous BRs regulates yield. Several studies have shown that exogenous BR treatment affects not only plant growth and development but also flowering, sex expression, and fruit development in cucurbits (Table 2). The application of EBL promoted the earlier appearance of the first female flower and increased the total number of female flowers on the main stem in a monoecious cucumber but not in a gynoecious cucumber. This increase was correlated with increased ET production, suggesting that the effect of BRs may be associated with ET biosynthesis in cucumber. However, in andromonoecious melon and monoecious zucchini, femaleness does not increase in response to EBL treatment [79]. Manzano et al. [100] reported that BRs may regulate the induction of female flowers in *C. pepo*, although this regulation is genotype-dependent. In addition, BRs may have relatively minor effects on sex expression in *C. pepo* compared with ET [100]. In another study, exogenous BR treatment influenced plant flowering and increased yield in watermelon [101]. Similar results were observed in cucumber. Exogenous BRs promote the vegetative growth and flowering of cucumber plants, increasing fruit yield [102,103]. Interestingly, Fu et al. [104] reported the effects of exogenous EBL application on fruit development, cell division, and the expression of cyclins and cyclin-dependent kinases (CDKs) in cucumber cultivars with different parthenocarpy capacities. The application of EBL in a cultivar without parthenocarpy induced cell division and parthenocarpic fruit growth, whereas BRZ treatment in a cultivar with parthenocarpy inhibited fruit set and, subsequently, growth. The application of EBL reversed this inhibitory effect. These findings indicate that BRs play a regulatory role in the early fruit development of cucumber plants [104].

BRs affect many physiological processes, including photosynthesis, the maintenance of the chloroplast structure, and the antioxidant defense system [105,106]. EBL treatment was associated with increased CO_2_ assimilation and increased quantum yield of PSII (ΦPSII) in cucumber. In contrast, BRZ treatment reduced plant growth and decreased CO_2_ assimilation and ΦPSII. Thus, the growth-promoting activity of BRs can be attributed to increased plant photosynthesis. Moreover, EBL upregulated the expression of genes encoding the small and large subunits of Rubisco, whereas BRZ downregulated the expression of these genes. In addition, EBL had a positive effect on Rubisco activity and increased the expression of genes encoding other proteins of the Calvin cycle. Thus, BRs can promote photosynthesis and growth by positively regulating the synthesis and activation of various photosynthesis-related enzymes in cucumber [107]. Another study reported the potential role of BRs in reducing the growth inhibition of cucumber plants under autotoxicity stress [105]. The application of EBL to cucumber leaves enhanced the phenotypic properties of the cucumber seedlings, which decreased under autotoxicity stress conditions. EBL treatment also improved the production of photosynthetic pigments, the photosynthetic rate, and stomatal opening while maintaining the integrity of chloroplasts. Increased activities of catalase (CAT), peroxidase (POD), ascorbate peroxidase (APX), and the antioxidative compound ascorbate (AsA) and reduced glutathione (GSH) contents were observed after EBL treatment. In contrast, the malondialdehyde (MDA) and ROS contents and the relative permeability of the cell membrane were reduced. These findings suggest that EBL application potentially plays a role in assisting phytoremediation and reducing autotoxicity stress in cucumber [105].

**Table 2:** The role of exogenous BRs in the growth and development of cucurbits.

Species	Role of BRs	Reference
*Cucumis sativus*	promoting earlier and increased female flower production in monoecious genotypes	[79]
	role in early fruit development and parthenocarpy	[104]
	promoting photosynthesis and growth by positive regulation of synthesis and activation of photosynthesis-related enzymes, including Rubisco	[107]
	promoting vegetative growth and yield	[102]
	role in the regulation of the antioxidant system and protection of the chloroplast under autotoxicity stress conditions	[105]
	increasing yield parameters	[103]
*Cucurbita pepo*	role in sex expression and flower development	[100]
*Citrullus lanatus*	increasing female flower production and yield parameters	[101]

## 8. BRs in the Stress Response of Cucurbits

Plants respond to various environmental stresses through physiological, biochemical, and molecular changes, which are often mediated by endogenous and exogenous phytohormones, including BRs. Genes involved in BR biosynthesis and signaling, especially those of the *BZR*, *BES1*, and *BAK1* families, play critical roles in modulating stress responses in cucurbits through complex regulatory networks (Table 3).

### 8.1. Abiotic Stress

In cucumber, BZR1 and BES1, two key transcription factors that act downstream of the BR signaling pathway, positively regulate the expression of BR-responsive genes and contribute to cold tolerance in cucumber seedlings [106,108]. Cold stress downregulated the expression of the BR biosynthetic genes *CsDET2* and *CsCYP90A1*, and this effect decreased more in the cucumber mutant of the *BASIC PENTACYSTEINE 2* (*CsBPC2*) gene, which encodes a transcription factor involved in the regulation of plant responses to phytohormones, including BRs. Interestingly, cold treatment led to increased expression of *CsBZR1, CsBZR2,* and cold-responsive genes, suggesting that *CsBPC2* knockout impacts both cold-responsive and BR biosynthetic genes [109].

*CsBZR* genes are also responsive to salt stress. Upon NaCl treatment, the expression of *CsBZR1*, *CsBZR2*, and *CsBZR3* increased more than 20-fold in cucumber seedlings, whereas the expression of other *CsBZR* genes increased 5–10-fold at 6 and 12 h after NaCl treatment but then decreased at 24 h [108]. In contrast, the *CsBES1* genes presented diverse expression patterns in the leaves and roots of cucumber seedlings after NaCl treatment [106]. Interestingly, the zucchini mutant *CsDWF5*, which is defective in BR biosynthesis, was less affected by salt stress. This was combined with greater upregulation of genes associated with salt tolerance, including those involved in abscisic acid (ABA) biosynthesis and signaling, calcium signaling, and those encoding cation exchangers and transporters [97].

The genes involved in BR signaling can respond to drought stress in cucurbits. In cucumber leaves, the expression of three *CsBES1* genes (*Csa1G467200*, *Csa2G361450*, and *Csa4G083490*), encoding BES1 transcription factors, significantly increased after 6 h of PEG treatment and then decreased. Moreover, the expression of two *CsBES1* genes (*Csa6G003450* and *Csa6G501930*) remained stable until 6 h, after which it decreased fourfold. In roots, the expression of all the *CsBES1* genes sharply increased from 9 to 24 h after PEG treatment [106]. In addition, the genes encoding the BZR transcription factors *CsBZR3*, *CsBZR4*, *CsBZR5*, and *CsBZR6* were upregulated under PEG-induced drought stress. Similarly, in *C. moschata*, *CmoBES1* genes are differentially expressed under salt, drought, and cold stresses, indicating the broad role of *CmoBES1* genes in abiotic stress responses [110]. Heavy metal stress, such as cadmium (Cd) stress, also influences *CsBZR* gene expression in cucumber. With the exception of *CsBZR4* and *CsBZR5*, all the other *CsBZR* genes were upregulated after 6 h of Cd exposure [108].

### 8.2. Biotic Stress

BR signaling appears to contribute to pathogen defense. *BAK1*, a coreceptor of *BRI1*, plays a vital role in BR signal transduction and response to environmental factors. In cucumber, the expression of *CsBAK1* gene family members changes after pathogen infection, suggesting specific roles for individual *CsBAK1* genes in defense against various pathogens. For example, *CsBAK1-14* expression was strongly induced or repressed by five major cucumber pathogens: gummy stem blight, powdery mildew, downy mildew, gray mold, and fusarium wilt. Specifically, the upregulation of *CsBAK1-14* expression was observed in susceptible lines after infection with these pathogens. These findings highlight the diverse and specialized functions of the *CsBAK1* gene family in the cucurbit response to pathogens [111]. Additionally, downregulation of one of the *CsBZR*s (*Csa1G524640*) was observed in cucumber in response to *Pseudomonas syringae* pv. *lachrymans*, which is a causal agent of angular leaf spot disease [112].

**Table 3:** The role of endogenous BRs in the stress response of cucurbits. Abbreviations: cv.—cultivar, d—days, dpi—days post-inoculation, h—hours, hpi—hours post-inoculation, R-line—resistant line, S-line—susceptible line.

Species	Investigated Accessions	Stress Factor	Exposure Time	Investigated Gene	Reference
*Cucumis sativus*	cv. Xintaimici	cold 6 °C	6, 12, 24 h, 2, 3, 6, and 9 d	*CsBES1*	[106]
		NaCl 150 mM	1, 3, 6, 9, 12, and 24 h		
		PEG6000 10%			
	cv. Changchunmici—wild type *Csbpc2*—knockout mutants	cold 4 °C	6 h	*CsBZR1* *CsBZR2* *CsCYP90A1* *CsDET2*	[109]
	cv. Xinchun 4	CdCl_2_ 200 μM	6, 12, and 24 h	*CsBZR*	[108]
		cold 12 °C/8 °C			
		NaCl 200 mM			
		PEG 6000 20%			
	Gy14 R-line B10 S-line	*Pseudomonas syringae* pv. *lachrymans*	1 dpi	*CsBZR*	[112]
	PI 183967 R-line 931 S-line	gummy stem blight	12 hpi	*CsBAK1*	[111]
	SSL508–28 R-line D8 S-line	powdery mildew	48 hpi		
	PI 197088 R-line cv. Vlaspik S-line	downy mildew	24 hpi		
	9110 Gt R-line 9930 S-line	gray mold and fusarium wilt	12, 48, and 96 hpi		
*Cucurbita moschata*	cv. TianMiyihao	cold (4 °C)	6 h	*CmoBES1*	[110]
		NaCl (150 mM)			
		PEG6000 (20%)			
*Cucurbita pepo*	MUCU16—wild type *dwfcp*—mutant	NaCl (200 mM)	16 h for seed germination	*CpDWF5*	[97]
		NaCl (100 mM)	72 h for seedling elongation		

## 9. Application of BRs to Alleviate Stress During Cucurbit Production

Numerous studies have demonstrated that exogenous application of BRs can mitigate the effects of abiotic and biotic stresses in cucurbit crops, including cucumbers, melons, zucchinis, watermelons, and bitter gourds [46,113,114,115]. Among the BRs, EBL, BL, and HBL are the most commonly used because of their bioactivity, stability, and commercial availability. In this context, the role of exogenous BRs in mitigating biotic and abiotic stresses has been explored in various studies (Table 4).

The application of EBL significantly increased cucumber seedling growth, chlorophyll content, photosynthetic capacity, the activities of antioxidant enzymes, and cellular redox states after cold treatment [81,116,117,118]. It also enhances the tolerance of cucumber plants to hypoxia [119]. Prestorage application of EBL to the surface of zucchini fruits significantly reduces the severity of chilling injuries, decreases weight loss, and delays yellowing, suggesting that EBL priming may be a feasible strategy to mitigate chilling damage to zucchini fruits during storage [120].

In melon, preharvest foliar application of EBL decreased postharvest fruit weight loss, ET emission, ascorbic acid, and antioxidant depletion rates during fruit storage [121]. The exogenous application of EBL reduces the impacts of high temperature on melon plants grown under greenhouse conditions and enhances their early fruiting [122]. Foliar application of EBL also increased the leaf area, root length, surface area, photosynthesis rate, stomatal conductance, transpiration rate, and chlorophyll content [121,123]. Therefore, soaking melon plants in a solution containing BR induced drought resistance, increasing their height, number of leaves, plant dry weight, and root–crown ratio under varying field capacity and water availability conditions [124]. Moreover, EBL pretreatment in *C. pepo* ameliorated the adverse effects of salt stress by reducing lipid peroxidation and the sodium content and increasing the content of γ-aminobutyric acid (GABA) [125].

Wei et al. [81] demonstrated that pretreatment with BL relieved stress-induced oxidative damage in cucumber seedlings exposed to cold, salt, and drought stresses and increased the capacity for ET biosynthesis and AOX activity. Moreover, pretreatment with EBL improved the growth, chlorophyll content, carbonic anhydrase activity, and photosynthetic efficiency of cucumber plants grown under combined salt and excess copper stress and further increased the activity of various antioxidant enzymes, i.e., CAT, POD, and superoxide dismutase (SOD), as well as the proline content at the 40-day growth stage [126]. The addition of EBL and ACC significantly mitigated the germination-suppressive effect of NaCl in the incubation medium with cucumber seeds. An increase in ET production was observed during seed germination, which was suppressed by salt stress, but this effect was attenuated by EBL application [80]. In addition, HBL, another bioactive analog of BRs, altered the antioxidant enzyme levels by increasing the SOD and POD levels and decreasing the MDA content. These results suggest that HBL can increase growth attributes, chlorophyll content, and antioxidant enzymes in cucumber plants under mild and high NaCl conditions [127]. Furthermore, the application of exogenous EBL promoted the growth of cucumber plants and relieved plant damage caused by Ca(NO_3_)_2_ and NaHCO_3_ stresses [128,129,130], as well as excess cadmium, copper, and zinc in cucurbits [126,131,132,133], and regulated the response of plants to Fe deficiency [134]. The interactions between different phytohormones influence a plant’s response to pathogens. EBL pretreatment of the roots or shoots of cucumber two-leaf stage seedlings before *Fusarium oxysporum* inoculation significantly reduced disease severity, improved plant growth, and reduced losses in biomass, regardless of the application method. EBL treatment significantly decreased pathogen development and induced the accumulation of ROS, flavonoids, and phenolic compounds and the activities of defense-related and ROS-scavenging enzymes. EBL application also triggered a slight increase in H_2_O_2_, followed by increases in the transcript levels of WRKY transcription factors and defense-related genes [135]. Xia et al. [136] demonstrated that foliar application of EBL only to cucumber primary leaves induced systemic tolerance to photooxidative stress in untreated upper and lower leaves. The systemic accumulation of H_2_O_2_ and the systemic induction of stress response genes were also observed. In addition, foliar treatment with EBL also increased root resistance to fusarium wilt [136]. Recently, Kang et al. [87] revealed that cucumber plants whose biosynthesis of BRs or GAs was impaired were more susceptible to *P. melonis*. However, increasing levels of endogenous BRs or exogenous application of EBL increased the resistance of cucumber plants to this pathogen [87].

Tao et al. [137] reported that EBL pretreatment of whole insusceptible zucchini plants reduced the accumulation of cucumber mosaic virus (CMV) in systemic leaves but not in inoculated leaves. The EBL-induced response to CMV is not accompanied by salicylic acid accumulation, but EBL treatment leads to increased accumulation of H_2_O_2_ during the early phase of CMV infection. These findings suggest that an SA-independent pathway may mediate antiviral immunity, whereas ROS may play positive roles, and exogenous BR application may hold potential for controlling CMV in susceptible crops [137]. In melons infected with *Pseudoperonospora cubensis*, EBL application at 2.0 mg/L significantly reduced the disease index and increased antioxidant activities to eliminate excessive ROS and MDA, thereby contributing to the stability of the intrinsic properties of the photosynthetic phenomenon and chlorophyll fluorescence parameters. Increased Rubisco activity was also observed (62.89%), which suggested a significant role for elevated carbon fixation and assimilation and the upregulated expression of regulatory genes linked with Rubisco activity and the PSII reaction process. This implies that the application of exogenous BRs enhances the modulation of the physiological indices of melon plants against downy mildew disease [138]. These results provide evidence that BRs can play roles in various stress responses, both to environmental stress and during plant–pathogen interactions in cucurbits.

**Table 4:** The role of exogenous BR application in mitigating the stress response in cucurbits. Abbreviations: BL—brassinolide, EBL—24–epibrassinolide, and HBL—28–homobrassinolide.

Species	Stress Factor	BRs Concentration and Type	Treatment Method	Reference
*Cucumis sativus*	Ca(NO_3_)_2_ 80 mM	0.1 μM EBL	foliar spraying	[128]
		1, 5, and 10 μM EBL	adding to the hydroponic medium	[131,132]
	cold 4 °C	1.0 μM BL	foliar spraying	[81]
	cold 10 °C/7 °C	0.1 μM EBL	foliar spraying	[116]
	cold 12 °C/8 °C	0.1 μM EBL	foliar spraying	[117]
	cold 14 °C	0.1 μM EBL	foliar spraying	[118]
	copper 100 mg·kg^−1^	0.01 μM EBL	foliar spraying	[126]
	ferrum deficiency	0.01, 0.1, and 0.5 μM EBL	adding to a solid medium	[134]
	hypoxia	1 μg·L^−1^ EBL	adding to the hydroponic medium	[119]
	salinity 60 and 120 mM NaCl	1, 3, and 5 μM HBL	foliar spraying	[127]
	salinity 150 mM NaCl	0.01 μM EBL	foliar spraying	[126]
	salinity 200 mM NaCl	1 μM BL	foliar spraying	[81]
	salinity 250 mM	5 μM EBL	seed soaking	[80]
	NaHCO_3_ 30 mM	0.2 μM EBL	adding to the hydroponic medium	[130]
	PEG6000 16%	1 μM BL	foliar spraying	[81]
	*Fusarium oxysporum*	0.1 and 0.2 μM EBL	adding to the hydroponic medium/foliar spraying	[135]
		0.2 μM EBL	foliar spraying	[136]
	*Phytophtora melonis*	100 μM EBL	foliar spraying	[87]
*Cucumis melo*	drought	0.05, 0.10, and 0.15 ppm BR	seed soaking	[124]
	heat 42/32 °C	0.05, 0.1, 0.5, 1, and 1.5 mg·L^−1^ EBL	foliar spraying	[123]
	heat 47 ± 3 °C	0.1, 0.2, and 0.3 mg·L^−1^ EBL	foliar spraying	[121,122]
	postharvest fruit chilling	0.1 mg∙L^−1^ EBL	preharvest foliar spraying	[121]
	*Pseudoperonospora cubensis*	0.5, 1, and 2 mg·L^−1^ BL	foliar spraying	[133]
*Cucurbita pepo*	NaCl 40 and 80 mM	0.01 and 0.1 μM EBL	adding to the hydroponic medium	[125]
	postharvest fruit chilling	0.1 μM EBL	fruit spraying	[120]
	cucumber mosaic virus (CMV)	0.2 μM EBL	foliar spraying	[137]
*Citrullus lanatus*	Zn 2.5, 5, and 10 mM	0.025, 0.05, 0.1, 0.2, and 0.5 μM EBL	foliar spraying	[133]
*Momordica charantia*	cold 8 °C	0.0001, 0.001. 0.01, 0.1, and 10 mg·L^−1^ EBL	foliar spraying	[115]

## 10. Perspectives and Concluding Remarks

The identification and cloning of genes responsible for altered growth in cucurbits is highly valuable for improving plant architecture. Cucurbit crops traditionally have long and trailing stems, requiring a large area for planting and high labor input for plantation management. Therefore, reducing shoot length is conducive to improving the production efficiency of cucurbits [12,139]. In comparison, bushy, dwarf, or semi-dwarf cultivars can lead to more efficient cultivation management, allowing higher planting density, lodging tolerance, lower water consumption, and easier harvest with considerable cost savings [140]. Moreover, the gradual reduction in arable land and higher labor costs mean that the long-stemmed architecture of cucurbit crops is no longer optimal for high-yield production. Thus, improving the growth architecture of cucurbit crops is desirable and will become a more important breeding goal in the coming years, especially when combined with increased tolerance to environmental stresses [139]. On the other hand, single gene mutations that affect plant architecture often result in changes in multiple organs, which is undesirable for fine-tuned control strategies in modern breeding [12]. The manipulation of BR biosynthesis or signaling often results in pleiotropic effects on plants and thus provides both opportunities and challenges for their application [141]. In practice, bushy, compact, dwarf, or semi-dwarf plant growth controlled by dominant alleles with no significant defects in sex expression, fertility, or fruit development would prove useful in commercial breeding [139]. To date, most of the mutations described in cucurbits that alter plant architecture are recessive, and stress tolerance has been tested in only single mutants. In addition, genetic resources suitable for improving plant architecture are currently limited, especially in cucumber but also in other cultivated cucurbits, thus hindering progress in breeding programs [142,143].

In this work, we present the current state of BR research in cucurbit crops. We summarized the genes involved in plant architecture traits related to BR biosynthesis (Table 1). Five of these genes have been identified as EMS-induced mutants, with one spontaneous mutation and one T-DNA insertion mutation. Mutations in *CsDET2*, *CsDWF1*, *CsDWF5*, *CsDWF7*, and *CsCYP85A1*, which are responsible for the dwarf phenotype in cucumber, have been identified [90,91,92,93,94]. The first BR biosynthesis mutant, *CpDWF5*, in zucchini has been reported [97]. The interaction between the *ClDUF21* and *ClDWF1* genes has been described in watermelon and confirmed by gene editing [99]. All these BR-deficient mutants presented typical phenotypes, including shortened internodes and petioles, few or no lateral branches, darkened and wrinkled leaves, and reduced fertility [90,91,92,93,94,97,99]. In cucumber, exogenous application of BL can only partially restore the phenotype of mutant plants [90,91,92,93,94]. BRs are likely synthesized and function within the same tissue or even within the same cell [144,145]. This finding is also consistent with studies suggesting that the inability of BL treatment to fully restore the defective phenotype is likely due to the limited absorption of exogenously applied BL by plants or other unknown mechanisms, such as an alternative route of BR biosynthesis or signaling unique to cucurbits [146].

The genes related to BR signaling can play important roles in stress responses, both in response to environmental stress and during plant–pathogen interactions in cucurbits (Table 3). The roles of the *CsBES1* and *CmoBES1* genes in response to cold, salinity, and drought have been reported [106,110]. In cucumber, *CsBZR1* and *CsBZR2* increase the tolerance of plants to cold [108,109]. *CsBZRs* can also increase drought, salinity, and cadmium tolerance in cucumber [108]. This finding is consistent with other studies showing that *BZR1* and *BES1* increase salt, drought, temperature, and heavy metal tolerance in *Arabidopsis*, tobacco, tomato, wheat, and cotton [147]. *CsBAK1* promotes the response to gummy stem blight, downy and powdery mildew, gray mold, and fusarium wilt in cucumber plants. The silencing of BZR6 promotes endogenous GA biosynthesis and compromises GA-mediated resistance against *P. melonis* infection [87]. Similarly, in rice, *OsBAK1* positively regulates tolerance to the fungal pathogens *Magnaporthe grisea* and *Magnaporthe oryzae*, as well as the bacterium *Xanthomonas oryzae* pv. *oryzae* [111]. In the grapevine, the expression of heterologous *VqSERK3/BAK1* in *Arabidopsis bak1-4* mutant lines increased resistance to powdery mildew [148]. However, only a few studies on the cucurbit family have investigated the potential roles of BR signaling-related genes in response to abiotic and biotic stresses. The role of BRs in the growth and development of cucurbits, as well as the crosstalk between BRs and other phytohormones, is poorly understood. Only a few studies have focused on the interaction of BRs and phytohormones after treatment with exogenous BRs or the inhibition of BR biosynthesis by BRZ application. The interactions between BRs and ET, GA, or AUX in cucumber have been demonstrated; however, these interactions have only been studied at the morphological, physiological, or biochemical level [79,80,81,87,88]. Understanding the mechanisms by which BRs regulate stress responses in cucurbits is a promising direction for future research.

**Figure 6:**
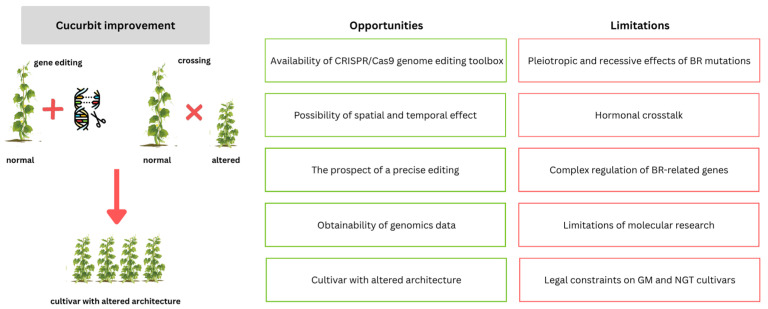
Strategy for improving plant architecture in cucurbits through brassinosteroid (BR)-related genetic engineering and classical breeding approaches. The scheme highlights key opportunities and limitations of BR-based strategies to improve cucurbits.

The CRISPR/Cas9 system has been shown to have great application value in the breeding of several crop species, including rice, maize, soybean, and tomato. Some successful attempts at gene editing related to plant architecture have also been reported in cucurbits [139,149,150]. For example, semi-dwarf plants have been designed for cucumber and loofah plants grown under greenhouse conditions to save labor. An upright bushy phenotype has been designed for pumpkins, watermelon, and melon to obtain relatively high yields per unit area under field conditions. This was achieved by editing the YABBY1 5′UTR in cucurbits, the protein translation of YABBY1, which is unique to angiosperms and has multiple functions in plant development and growth [139]. In other studies, the CRISPR/Cas9 system has been used to edit homologs of the ERECTA family of receptor-like kinase genes. This approach resulted in a compact plant architecture with shorter internodes in melon, squash, and cucumber [149]. Chen et al. [150] obtained a CRISPR/Cas9 mutation in the *CsIAGLU* gene encoding IAA glucosyltransferase. Mutants produce a greater level of IAA, resulting in an expansion of the cells on the adaxial side of the petiole base, thus forming a greater leaf pedicle angle [150]. In this way, CRISPR/Cas9 can be successfully used for precise breeding to improve plant growth architecture and other important traits. This study also provides an opportunity for the functional characterization of genes related to BR metabolism and their role in cucurbit growth to develop and advance BR-based technologies. BR metabolism-related genes may be future targets of the CRISPR/Cas9 strategy to obtain plants with improved architecture and other important utility traits in cucurbit breeding (Figure 6). Owing to the correlation between low BR levels and reduced fertility in cucurbit plants, the temporal and spatial regulation of Cas9 expression should be considered. This can be achieved by using tissue-specific or inducible promoters to increase the efficiency of targeted mutagenesis and reduce off-target effects [151].

**Figure 7:**
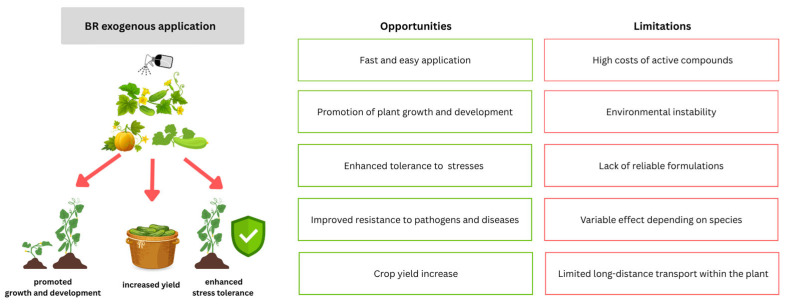
Strategy for improving plant growth and development, enhancing stress tolerance and disease resistance, and increasing yield in cucurbit crops through the exogenous application of brassinosteroids (BRs). The schematic illustrates the main opportunities and limitations of this approach.

This overview also highlights the impact of exogenous treatment with BRs on the growth and development of cucurbits (Figure 7). The specific effects of BRs on cucurbits include promoting vegetative and generative growth, enhancing female flower and yield production, and regulating the antioxidant system, photosynthesis, and chloroplast function (Table 2). The effects of BRs on reproductive development have also been shown in other species. For example, the number of ovules increased after the exogenous application of BL to Micro-Tom tomato plants harboring a mutation in the *DWF4* gene [152]. The exogenous application of EBL causes tomato fruit softening, whereas the application of BZR has the opposite effect, resulting in the accumulation of carotenoids in tomato fruits [153]. The increased tolerance to abiotic and biotic stresses after exogenous BR treatment has been confirmed in tomato, pepper, bean, soybean, radish, canola, rice, wheat, corn, and many other plants [114]. The increased tolerance of cucurbit plants to cold, heat, drought, salinity, and metal toxicity, with the action of BRs, has been proven via several morphological, physiological, biochemical, and molecular studies. The role of BRs in enhancing the defense response to biotic stress induced by various pathogens in cucumber, melon, and zucchini has also been demonstrated [87,135,136,137,138]. All these studies demonstrate how stresses cause damage and how BRs help cucurbit plants in the dynamic defense response to such pressures (Table 4). Given their wide spectrum effectiveness for every aspect of plant growth, even a modest increase of 10–15% could increase the gross annual productivity by 10–15 million tons [102]. Thus, elucidating the exact transport pathway of exogenously applied BR and its stability inside the cell may have a positive impact on the tolerance of cucurbits to abiotic and biotic stresses. In addition, exploring the impact of exogenous BRs on the biosynthesis and signaling of other phytohormones in response to stress is also a promising area of research. This knowledge may facilitate the integration of agronomy and molecular biology in the future to create a comprehensive network that links the phenotypic changes induced by BR application with the corresponding molecular changes.

Although BRs play an important role in plant developmental processes, they are difficult to apply in agriculture on a relatively large scale because of their high cost. Therefore, more suitable and cost-effective formulations of BRs in field and greenhouse crop production are needed [154]. Novel nanoformulated BRs or cost-effective BR analogs can help deliver solutions for horticultural practices to be applied in cucurbit production. Future research should focus on integrating cost-effective BR application strategies with gene-editing approaches to develop climate-resilient, high-yielding cucurbit cultivars suitable for diverse agricultural systems.

## Data Availability

No new data were created or analyzed in this study. Data sharing does not apply to this article.

## Abbreviations

The following abbreviations are used in this manuscript:

14-3-3	14-3-3 proteins
22-OH-CR	22-alpha-hydroxycampesterol
22,23-diOH-CR	22,23-dihydroxycampesterol
22-OH-3-one	22-alpha-hydroxy-5-alpha-campestan-3-one
22-OH-4-en-3-one	22-alpha-hydroxy-campest-4-en-3-one
24-MC	24-methylene cholesterol
26-OH-BL	26-hydroxybrassinolide
28-norCS	28-norcastasterone
3-dehydro-TE	3-dehydroteasterone
3-epi-6-deoxo-CT	3-epi-6-deoxocathasterone
campest-4-en-3-one	campest-4-en-3-one
3-dehydro-6-deoxo-TE	3-dehydro-6-deoxoteasteroneteasterone
6-oxo-CN	6-oxocampestanol
6-deoxo-CS	6-deoxocastasterone
6-deoxo-CT	6-deoxocathasterone
6-deoxo-TE	6-deoxoteasterone
6-deoxo-TY	6-deoxotyphasterol
ABA	abscisic acid
ACC	1-aminocyclopropane-1-carboxylic acid
ACO	ACC oxidase
ACS	ACC synthase
APX	ascorbate peroxidase
ARF	auxin response factor
AsA	ascorbate
AOA	aminooxyacetic acid
AUX	auxin
AVG	aminoethoxyvinylglycine
BAK1	BRI1-ASSOCIATED KINASE 1
BAS1	PHYB ACTIVATION TAGGED SUPPRESSOR 1 (also known as CYP734A1)
BES1	BRI1-EMS-SUPPRESSOR 1
BIN2	BRASSINOSTEROID INSENSITIVE 2
BKI1	BRI1 KINASE INHIBITOR 1
BL	brassinolide
BR	brassinosteroid
BR6OX1	BRASSINOSTEROID-6-OXIDASE 1 (also known as CYP85A1)
BR6OX2	BRASSINOSTEROID-6-OXIDASE 2 (also known as CYP85A2)
BRC1	BRANCHED1
BRI1	BRASSINOSTEROID INSENSITIVE 1
BRZ	brassinazole
BSK1	BR-SIGNALING KINASE 1
BSU1	BRI1 SUPPRESSOR 1
BZR1	BRASSINOZOLE-RESISTANT 1
CAT	catalase
CDG1	CONSTITUTIVE DIFFERENTIAL GROWTH 1
CDK	cyclin-dependent kinase
ClDUF21	*Citrulus lanatus* protein with domain of unknown function 21
ClDWF1	*Citrulus lanatus* C24 reductase
CMV	cucumber mosaic virus
CN	campestanol
CpDWF5	*Cucurbita pepo* 7-dehydrocholesterol reductase
CpTINY4	*Cucurbita pepo* TINY4
CPD	CONSTITUTIVE PHOTOMORPHOGENIC DWARF (also known as CYP90A1)
*cpa*	*compact*
*cpa-2*	*compact-2*
CR	campesterol
CSA	CARBON STARVED ANTHER
CS	castasterone
CsAOX	cucumber alternative oxidase
CsACS1	cucumber ACC synthase 1
CsACS2	cucumber ACC synthase 2
CsACS3	cucumber ACC synthase 3
CsACO1	cucumber ACC oxidase 1
CsACO2	cucumber ACC oxidase 2
CsBAK1	cucumber BRI1-ASSOCIATED KINASE 1
CsBES1	cucumber BRI1-EMS-SUPPRESSOR 1
CsBPC2	cucumber BASIC PENTACYSTEINE 2
CsBZR	cucumber BRASSINOZOLE-RESISTANT
CsBZR1	cucumber BRASSINOZOLE-RESISTANT 1
CsBZR2	cucumber BRASSINOZOLE-RESISTANT 2
CsBZR3	cucumber BRASSINOZOLE-RESISTANT 3
CsBZR4	cucumber BRASSINOZOLE-RESISTANT 4
CsBZR5	cucumber BRASSINOZOLE-RESISTANT 5
CsBZR6	cucumber BRASSINOZOLE-RESISTANT 6
CsCYP85A1	cucumber BR C6 oxidase
CsDET2	cucumber steroid 5-alpha reductase
CsDWF1	cucumber sterol C24 reductase
CsDWF5	cucumber 7-dehydrocholesterol reductase
CsDWF7	cucumber delta7-sterol C5 reductase
CsIAGLU	cucumber IAA glucosyltransferase
CT	cathasterone
CYP724B1	BR C22 hydroxylase (also known as DWF11)
CYP734A1	BR C26 hydroxylase (also known as BAS1)
CYP85A1	BR C6 oxidase (also known as BR6OX1)
CYP85A2	BR C6 oxidase (also known as BR6OX2)
CYP90A1	BR C3 oxidase (also known as CPD)
CYP90B1	BR C23 hydroxylase (also known as DWARF4)
CYP90C1	BR C23 hydroxylase (also known as ROT3)
CYP90Ds	BR C23 hydroxylases
DET2	steroid-5-alpha reductase (also known as DWARF6)
DWF1	DWARF1 sterol C24 reductase
DWF4	DWARF4 (also known as CYP90B1)
DWF5	DWARF5 5,7-sterol-7-reductase
DWF6	DWARF6 (also known as DET2)
DWF7	DWARF7,7-sterol-C5-desaturase
DWF11	DWARF11 (also known as CYP724B1)
dwfcp	DWARF in *Cucurbita pepo*
EBL	24-epibrassinolide
ERECTA	receptor-like kinase (RLK)
ET	ethylene
GA	gibberellin
GA20ox1	GA20 oxidase 1
GABA	gamma-aminobutyric acid
GPOX	guaiacol peroxidase
GR	glutathione reductase
GSH	reduced glutathione
GSK3	glycogen synthase kinase 3
GST	glutathione-S-transferase
HBL	28-homobrassinolide
HG	homogalacturonan
IAA	indole-3-acetic acid
KAO	kaurenoic acid oxidase
KIB1	KINK SUPPRESSED IN BZR1-1D
LRR-RLK	protein kinase with leucine-rich repeats
MDA	malondialdehyde
MAPK	mitogen-activated protein kinase
NaCl	sodium chloride
PEG	polyethylene glycol
POD	peroxidase
PP2A	PROTEIN PHOSPHATASE 2A
ROS	reactive oxygen species
ROT3	ROTUNDIFOLIA 3 (also known as CYP90B1)
*scp-1*	*super compact-1*
*scp-2*	*super compact-2*
*scp-3*	*super compact-3*
SERK	SOMATIC EMBRYOGENESIS RECEPTOR-LIKE KINASE
SHAM	salicylhydroxamic acid
SOD	superoxide dismutase
TCP	TEOSINTE BRANCHED1/CYCLOIDEA/PROLIFERATING CELL FACTORS
TE	teasterone
TY	typhasterol
YABBY1	transcription factor YABBY1
